# Assessment of Population Immunity to Enteric Hepatitis Viruses in the Population of Belgrade

**DOI:** 10.3390/epidemiologia7030072

**Published:** 2026-05-22

**Authors:** Anna Yurievna Popova, Alesia Yuryevna Olkhovskaya, Luka Dragačević, Yulia Vladimirovna Ostankova, Svetlana Alexandrovna Egorova, Alexander Nikolaevich Schemelev, Darya Tsibulskaya, Ekaterina Vladimirovna Anufrieva, Anastasiya Romanovna Ivanova, Irina Victorovna Drozd, Ojuna Bayarovna Zhimbaeva, Branko Beronja, Jelena Protić, Ekaterina Mikhailovna Danilova, Angelica Marsovna Milichkina, Valeri Andreevich Ivanov, Oleg Vladimirovich Kotsar, Edward S. Ramsay, Vyacheslav Yurievich Smolensky, Areg Artemovich Totolian

**Affiliations:** 1 Federal Service for the Oversight of Consumer Protection and Welfare, 127994 Moscow, Russia; popovaay@pasteurorg.ru (A.Y.P.); smolenskiy_vy@rospotrebnadzor.ru (V.Y.S.); 2 Saint Petersburg Pasteur Institute, 197101 St. Petersburg, Russia; alesja.gorbynova@gmail.com (A.Y.O.); shenna1@yandex.ru (Y.V.O.); egorova@pasteurorg.ru (S.A.E.); kate.an21@yandex.ru (E.V.A.); shinvy@inbox.ru (A.R.I.); div-o@mail.ru (I.V.D.); zhimbaeva@pasteurorg.ru (O.B.Z.); emdanilova@pasteurorg.ru (E.M.D.); amilichkina@pasteurorg.ru (A.M.M.); korsaring@yandex.ru (V.A.I.); kotsar@pasteurorg.ru (O.V.K.); totolian@spbraaci.ru (A.A.T.); 3 Institute of Virology, Vaccines, and Sera “Torlak”, 11152 Belgrade, Serbia; ldragacevic@torlak.rs (L.D.); dtsibulskaya@torlak.rs (D.T.); jprotic@torlak.rs (J.P.); 4 Clinic for Infectious and Tropical Diseases, University Clinical Center of Serbia, 11000 Belgrade, Serbia; brankoberonj99@gmail.com

**Keywords:** hepatitis A, hepatitis E, seroprevalence, Serbia, epidemiology, population immunity profile

## Abstract

Background: Hepatitis A (HA) and E (HE) represent a significant global health burden. Despite the development of effective vaccines against hepatitis A virus (HAV) and hepatitis E virus (HEV), outbreaks of acute HA and HE continue to occur worldwide. This study aimed to assess the seroprevalence of anti-HAV and anti-HEV IgG antibodies (Abs) in the population of Belgrade and to analyze their association with socio-demographic and clinical factors. Materials and Methods: A cross-sectional study was conducted on a sample of 2533 healthy volunteers in Serbia in May 2024. Participation was voluntary and web-based, leading to an overrepresentation of women and middle-aged adults, while children were underrepresented. Due to this non-probabilistic recruitment, the absolute seroprevalence estimates have limited generalizability to the entire population of Belgrade. Serum samples were tested for anti-HAV and anti-HEV IgG using commercial ELISA kits. The anti-HEV estimate is based on a single ELISA without confirmatory testing and should be interpreted with this limitation in mind. Statistical analysis included confidence interval estimation, chi-square tests, and Spearman’s correlation. Results: Overall seroprevalence was 20.5% (95% CI: 18.9–22.1) for anti-HAV and 22.6% (95% CI: 21.0–24.3) for anti-HEV. A strong, non-linear increase in anti-HAV seroprevalence with age was observed, rising sharply from 2.8% in the 18–29 group to 78.3% in those aged 70+. Anti-HEV seroprevalence also featured a significant positive correlation with age (rs = 0.99, *p* < 0.0001), increasing from 4.2% in children (1–17 years) to 49.2% in the 70+ group. Men had significantly higher anti-HAV seroprevalence than women (23.1% vs. 19.3%, *p* = 0.029). Individuals with a history of surgical interventions or blood transfusions had significantly higher odds of being anti-HEV positive (OR = 1.41, *p* = 0.0005). Vaccination coverage against HAV was low (1.8%), and Abs were detected in only 28.6% of vaccinated individuals. Conclusions: This study suggests high HEV seroprevalence and an age-polarized HAV seroprevalence in Serbia, indicating a significant shift in the epidemiological landscape while acknowledging the sampling and assay limitations stated above. The findings underscore a growing population susceptible to HAV and highlight the need for reinforced vaccination strategies, improved diagnostics, and targeted public health interventions.

## 1. Introduction

Due to wide geographic distribution, high incidence rates, and a lack of specific therapies, the problem of enteric hepatitis remains relevant today. According to a 2015 WHO report, hepatitis A caused an estimated 11,000 deaths worldwide in 2015, while hepatitis E caused 44,000 deaths (WHO 2017) [1]. Both viruses are primarily transmitted via the fecal–oral route, affecting the liver and causing acute inflammatory processes. These are manifested as symptoms of intoxication, impaired liver function, and jaundice [2,3]. The first cases of viral hepatitis became known to physicians in ancient times. Even then, doctors noted a connection between disease symptoms and consumption of contaminated water or food. However, scientific understanding of the nature and mechanism of these diseases only emerged at the end of the 19th century.

Hepatitis A and E (HA, HE) are predominantly prevalent in developing countries with low levels of sanitation and water supply, where they pose significant public health challenges leading to mass epidemics and serious economic consequences [4,5]. Hepatitis E is particularly dangerous for pregnant women as it causes severe illness with a high risk of maternal and perinatal mortality [6]. Importantly, both HA [7] and HE [8] are recognized precipitants of acute-on-chronic liver failure (ACLF) in patients with advanced chronic liver disease (ACLD). The incidence of ACLF among patients with ACLD is estimated at 24–40 per 1000 patient-years, and mortality remains high, with a 28-day fatality of 30–50%, and 90-day mortality exceeding 50–70%, often even higher when ACLF is triggered by acute viral hepatitis. Effective control measures include various sanitary-hygienic interventions, increased population immunization, and improved water supply and sanitation conditions. These actions are crucial for reducing the burden on healthcare systems and economies of countries affected by epidemics caused by the hepatitis A virus (HAV) and hepatitis E virus (HEV) [9].

According to national infectious disease reports, a total of 13,746 HA cases were registered in Serbia for the period between 2002 and 2023 [10]. Average incidence declined dramatically from approximately 14.8 per 100 K population for the period 2002–2006 to only 0.8 per 100 K in 2017–2023. A significant shift in age distribution was observed, with peak incidence transitioning from children aged 5–9 years to adolescents aged 10–19 years during 2017–2023. In recent years, person-to-person transmission has predominated, while water- or foodborne outbreaks (fecal–oral route) have become uncommon. A total of six deaths attributable to HAV infection were recorded, corresponding to a mortality rate of 0.07 per 100 K population. The number of outbreaks declined substantially from 174 in the early 2000s to only 16 during 2017–2023.

In Serbia, HE remains substantially under detected within the national surveillance system. Only seven HEV cases were officially reported between 2022 and 2024, a figure that more likely reflects limited diagnostic use rather than the true incidence of infection [10]. Population-level data are limited; the most recent national seroepidemiological study (2014) reported a 15% anti-HEV IgG seroprevalence among blood donors, indicating notable past exposure despite the absence of routine testing [11]. Animal reservoir studies support ongoing zoonotic circulation of HEV genotype 3. Investigations in domestic swine consistently find 30–40% seroprevalence, with 10–29% HEV RNA positivity in liver samples of young pigs at slaughter, confirming active viral transmission in the pork production chain [12,13]. Collectively, these epidemiological indicators strongly suggest that the actual burden of HEV infection in Serbia is underestimated and likely exceeds the number of clinically detected cases.

Despite the development of effective vaccines against HAV and HEV, outbreaks of acute hepatitis A and E continue to occur worldwide. Most patients with acute HA and HE recover spontaneously. However, in some cases, adults develop acute hepatic failure due to immune-mediated liver damage [14,15]. HEV, especially genotypes 3 and 4, can lead to chronic hepatitis and fibrosis in individuals with weakened immunity, while infections with genotype 1 in developed countries may feature a severe course, including those during pregnancy [15].

By virtue of the currently available vaccine against hepatitis E, HEV 239 (Hecolin), there is now an opportunity to protect vulnerable populations from HE and control disease outbreaks. This vaccine was registered in China in 2011 and is intended for vaccination of healthy adults aged 16 years or older through intramuscular administration according to a three-dose schedule (0, 1, and 6 months) [16,17].

In March 2022, a significant event took place in the field of HE vaccination on the African continent. In response to an infection outbreak in Bentiu (South Sudan), a vaccination campaign against HE was conducted. More than 72,000 people were vaccinated, including pregnant women [18]. This was the first historical case of using a vaccine to eliminate the consequences of an outbreak. A second similar campaign was held in 2023 in response to an outbreak in Fangak County, Jonglei Province, South Sudan [19].

Today, more than seven vaccines already exist against HAV, among them: Algavac and Algavac M (Russia); Avaxim (France); Vaqta (USA); Havrix (Belgium); Healive (China); and Aimmugen (Japan) [20]. The vaccination scheme against HAV usually includes two intramuscular injections. Risk groups for whom HA vaccination is recommended include: those living in regions with unfavorable HA morbidity; those exposed to occupational risk of infection and capable of becoming spreaders (medical workers, service industries workers, food industry workers, those involved with servicing water or sewage networks); those traveling to unfavorable countries or regions where outbreak-related HA morbidity has been recorded; and contacts in HA foci [21].

The aim of our work was to assess the prevalence of antibodies (Abs) to HAV and HEV in the Republic of Serbia, followed by ranking based on infectious-vaccination status and socio-demographic characteristics.

## 2. Materials and Methods

### 2.1. Study Design and Ethics Approval

A cross-sectional study, “Herd immunity to vaccine-preventable and other relevant infections in the Belgradian population”, was conducted in May 2024 under a joint program between Rospotrebnadzor (Russia) and the Serbian Ministry of Health. It was approved by the local ethics committees at the Saint Petersburg Pasteur Institute (St. Petersburg, Russia) and the Institute of Virology, Vaccines, and Sera “Torlak” (Belgrade, Serbia). The ethics approval documents are available from the corresponding author upon request.

### 2.2. Participant Recruitment and Randomization

Selection of volunteers was carried out using a web application (Saint Petersburg Pasteur Institute, St. Petersburg, Russia) [22,23,24]. Before the study, all participants, or their legal representatives, were familiarized with the purpose and methodology of the study and signed a statement of informed consent. Volunteers for the study were selected through a survey questionnaire. The questionnaire collected personal information including full name, gender, date of birth, address, occupation, school attendance (for children) or university studies, contact details (phone number, email), and medical institution of volunteer registration. Information was also collected regarding chronic diseases, blood transfusions, and surgical interventions (with dates). Specific information related to each infection included in the study (HA, HE) was collected: whether they had experienced illness and corresponding dates; and whether HAV vaccination or booster vaccinations were administered (specifying vaccine names and dates). Information on vaccinations and disease history was collected from vaccination certificates or other medical documentation when possible, and only those with documented proof were considered as vaccinated or having had hepatitis A. After questionnaire processing, individuals meeting the established criteria were invited to a medical center for subsequent laboratory testing. This recruitment method constitutes non-probabilistic (convenience) sampling, as participation was entirely voluntary. Age groups were predefined based on census data to mirror the age distribution of Belgrade’s population. However, quotas were not enforced for sex or other characteristics, and participation remained voluntary. This limits the generalizability of absolute seroprevalence estimates.

### 2.3. Inclusion and Exclusion Criteria

Participation in the study was entirely voluntary. The primary inclusion criterion was the provision of valid written informed consent at the blood collection point. Key exclusion criteria were: (1) the inability or unwillingness to provide such consent; (2) the presence of an acute illness at the time of the visit; or (3) current receipt of immunosuppressive therapy. These medical exclusions were implemented to minimize confounding effects on serological markers. All individuals meeting the requirements (selected via the web application, providing final written consent at the collection point, meeting the inclusion criteria, completing study procedures) were enrolled.

### 2.4. Study Population and Sampling

#### 2.4.1. Sample Size Calculation and Stratification

The representative sample size was calculated using a formula based on the central limit theorem, utilizing an online calculator [25], as described previously [22,23,24]. In addition, the sampling framework was based on official census data for the population of Belgrade published by the Statistical Office of the Republic of Serbia (2011 Census, with updated population estimates) [26]. These data were used to ensure adequate representativeness across predefined age groups. The predefined age stratification was intentionally designed to reflect standard demographic and epidemiological age categories commonly used in national surveillance and international seroprevalence studies.

#### 2.4.2. Study Sample Size and Demographics

The studied sample comprised 2533 healthy volunteers stratified into nine age groups: 1–5 years old (*n* = 13, 0.5%), 6–11 years old (*n* = 43, 1.7%), 12–17 years old (*n* = 62, 2.4%), 18–29 years old (*n* = 249, 9.8%), 30–39 years old (*n* = 501, 19.8%), 40–49 years old (*n* = 688, 27.2%), 50–59 years old (*n* = 468, 18.5%), 60–69 years old (*n* = 320, 12.6%), and ≥70 years old (*n* = 189, 7.5%). It should be noted that the male portion of the surveyed volunteer sample accounted for 32.3% (*n* = 819), while the female portion made up 67.7% (*n* = 1714) [24].

#### 2.4.3. Consideration of Potential Sampling Bias

Although the sample included a higher proportion of female participants (67.7%), this imbalance most likely reflects voluntary participation patterns and healthcare-seeking behavior, which has been consistently reported in population-based seroepidemiological studies. Importantly, hepatitis A and E serological markers are not considered to be strongly sex-dependent. Therefore, this imbalance is unlikely to have significantly biased the main outcomes of the study.

#### 2.4.4. Serological Testing

ELISA testing for the presence of HA and HE markers involved qualitative determination of anti-HAV and anti-HEV IgG Abs by commercial assays (JSC “Vector-Best”, Novosibirsk and NPO “Diagnostic Systems”, Nizhny Novgorod, Russia) following manufacturer instructions. Both test systems include manufacturer-provided positive and negative controls (anti-HAV: calibrators 0, 20, and 200 mIU/mL; anti-HEV: K+ and K−).

#### 2.4.5. Statistical Analysis

Statistical processing was carried out using the software packages MS Excel and GraphPad Prizm 9.3 (GraphPad Software Inc., San Diego, CA, USA, https://www.graphpad.com/support/prism-5-updates/ (accessed on 15 November 2025)). To estimate statistical error, we used the exact Clopper–Pearson interval. Results are presented with a 95% confidence interval (95% CI). For assessing the significance of differences in numerical data obtained from paired comparisons, depending on sample characteristics, either Fisher’s exact test or Yates-corrected chi-square test was applied. To account for multiple comparisons, the Bonferroni correction was applied where appropriate. Correlation analysis was performed based on compliance with parametric distribution by calculating Spearman’s rank correlation coefficient (r_s_), which was evaluated according to the Chaddock scale. For graphical illustration only, linear and exponential trend lines were fitted to age-group aggregated seroprevalence data using least-squares regression. The coefficient of determination (R^2^) was calculated to describe the variance explained by the model. These analyses are based on grouped data and may overestimate the strength of association. Differences were considered statistically significant when *p* < 0.05.

## 3. Results

### 3.1. Overall Seroprevalence of Anti-HAV and Anti-HEV Antibodies

**Figure 1:**
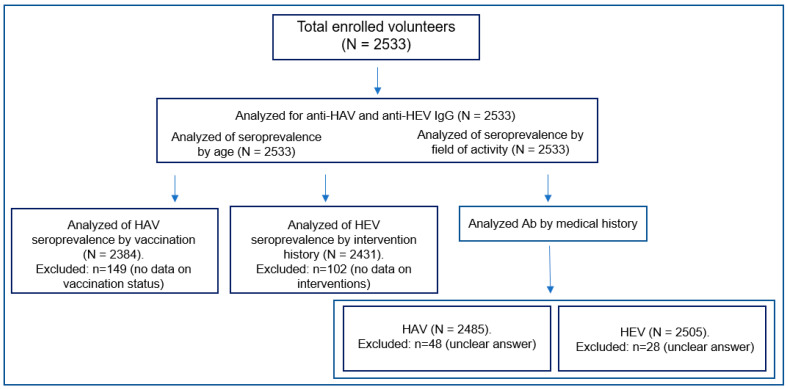
Flow chart of the study population selection and analysis.

A total of 2533 volunteers were enrolled and tested for the presence of anti-HAV and anti-HEV IgG antibodies. Figure 1 illustrates the selection of the study population and the formation of subgroups for subsequent analyses.

Over the course of the study, anti-HAV Abs were detected in 519 out of 2533 individuals, which corresponds to 20.5% (95% CI: 18.9–22.1). Anti-HEV Abs were found in 572 (of 2533), which corresponds to 22.6% (95% CI: 21.0–24.3). In addition, Abs against both HAV and HEV were identified in 179 subjects, accounting for 7.1% (95% CI: 6.1–8.1).

Comparative analysis based on gender revealed that anti-HAV seroprevalence was higher among males (*n* = 189 out of 819, 23.1%, 95% CI: 20.2–26.1) than among females (*n* = 330 out of 1714, 19.3%, 95% CI: 17.4–21.2). This difference was significant (χ^2^ = 4.742, *p* = 0.0294, df = 1, OR = 1.258, 95% CI: 1.0–1.5). However, no statistically significant differences were observed regarding HEV Ab prevalence between males (*n* = 192 out of 819, 23.4%, 95% CI: 20.6–26.5) and females (*n* = 380 out of 1714, 22.2%, 95% CI: 20.2–24.2; *p* > 0.05).

### 3.2. Age-Specific Seroprevalence of HAV and HEV

Seroprevalence (HAV, HEV) was assessed among various age groups (Table 1). No statistically significant differences were found when comparing HAV Ab seroprevalence across three pediatric age groups (1–5, 6–11, 12–17 years) or three adult age groups (18–29, 30–39, 40–49 years) (*p* > 0.05).

However, a comparison of older age groups showed significantly higher Ab prevalence in those aged 70+ years compared to the group aged 60–69 years (χ^2^ = 29.652, *p* < 0.0001, df = 1, OR = 3.106, 95% CI: 2.1–4.7) and also compared to the group aged 50–59 years (χ^2^ = 180.01, *p* < 0.0001, df = 1, OR = 12.953, 95% CI: 8.6–19.5). Additionally, HAV Ab seroprevalence in the group aged 60–69 years was significantly higher than in the group aged 50–59 years (χ^2^ = 84.161, *p* < 0.0001, df = 1, OR = 4.170, 95% CI: 3.1–5.7). Furthermore, the presence of anti-HAV Abs in all groups aged ≥50 years (50–59, 60–69, 70+ years) was significantly higher compared to each of the younger groups under 50 years old (*p* < 0.05).

When comparing the distribution of anti-HEV Abs across different pediatric age groups, no significant differences were found (*p* > 0.05). Similarly, there were no significant differences between children’s groups and adults aged 18–29 years or 30–39 years (*p* > 0.05).

**Figure 2:**
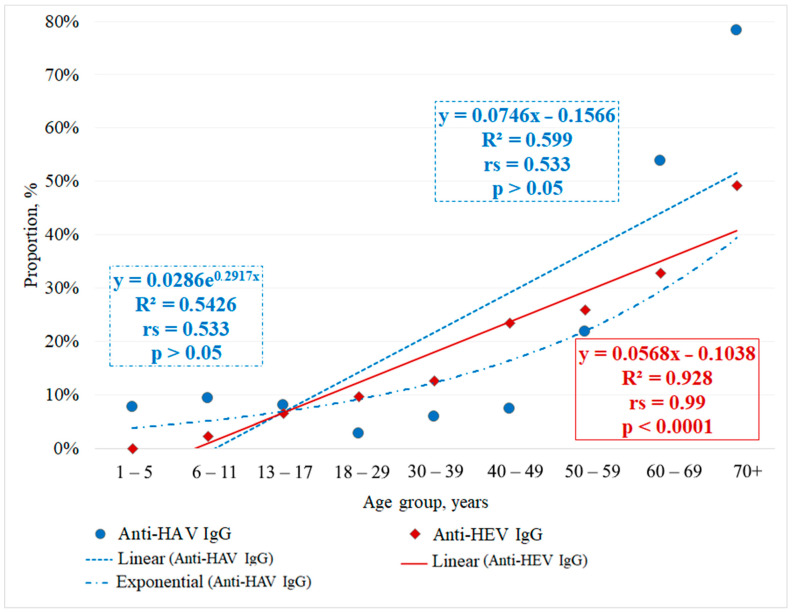
Prevalence of anti-HAV and anti-HEV IgG antibodies by age group.

Due to a lack of differences between the two middle-age groups (40–49, 50–59 years), yet significant differences relative to other groups, these two groups were combined into one (40–59 years). HEV Ab seroprevalence in this merged group (40–59 years) was significantly higher compared to the group aged 30–39 years (χ^2^ = 28.906, *p* < 0.0001, df = 1, OR = 2.243, 95% CI: 1.7–3.0) and significantly lower compared to the group aged 60–69 years (χ^2^ = 8.751, *p* = 0.0031, df = 1, OR = 1.514, 95% CI: 1.2–2.0). Moreover, HEV Ab seroprevalence in the group aged 60–69 years was significantly lower compared to the group aged 70+ years (χ^2^ = 12.755, *p* = 0.0004, df = 1, OR = 1.984, 95% CI: 1.4–2.9). The correlations between Ab occurrence (anti-HAV, anti-HEV) and participant age were assessed (Figure 2).

Within the analysis, we determined the trend line for HAV Ab seroprevalence as a function of age. No linear dependence was established between HAV seroprevalence and age: coefficient of determination (R^2^) = 0.599; multiple correlation coefficient (R) = 0.77; Spearman rank correlation coefficient (r_s_) = 0.533; degrees of freedom (df) = 7; critical value of the Spearman criterion = 0.7; *p* > 0.05; and strength of association (Chaddock scale)—moderate. However, a strong positive non-linear relationship was observed between HAV Ab seroprevalence and age, consistent with the well-documented cumulative risk of infection over a lifetime. When considering the relationship as exponential, the coefficient of determination (R^2^) was 0.8552, and the multiple correlation coefficient (R) was 0.92. The R^2^ values presented in Figure 2 are derived from aggregated age-group data and are presented for descriptive illustration only; they may not reflect individual-level variability.

For the group aged 30 years and over separately, the following values were calculated: Spearman’s rank correlation coefficient r_e_ = 0.99; degrees of freedom df = 3; critical value of Spearman’s criterion = 1; *p* < 0.05; and strength of association (Chaddock scale)—very strong.

A correlation between the prevalence of anti-HEV Abs and age was identified. When considering this relationship as linear, the following characteristics were defined: coefficient of determination (R^2^) = 0.928; multiple correlation coefficient (R) = 0.96; Spearman rank correlation coefficient (r_s_) = 0.99; df = 7; critical value of the Spearman criterion = 0.7; *p* < 0.0001; and strength of association (Chaddock scale) = very strong.

### 3.3. Agreement Between Self-Reported History of Hepatitis and Seropositivity

Comparison of laboratory-confirmed seropositivity with documented medical history revealed a discrepancy, highlighting a substantial proportion of unrecognized or asymptomatic infections.

**Figure 3:**
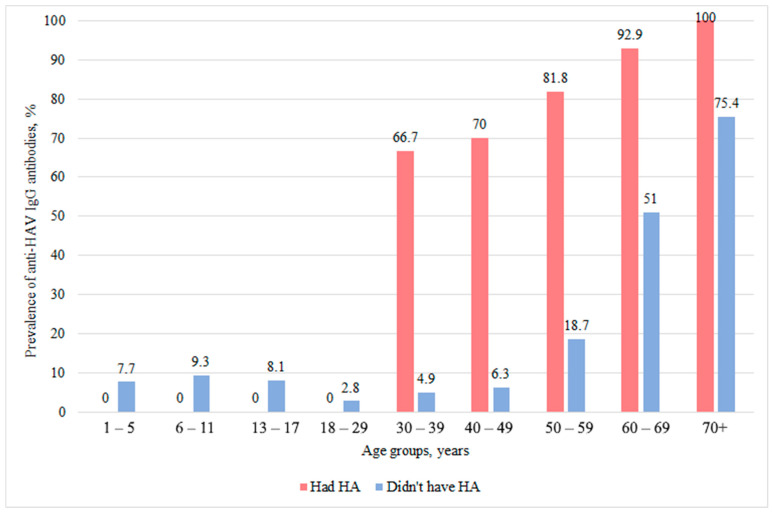
Anti-HAV IgG seroprevalence by age and hepatitis A history.

Of the 2533 volunteers, 48 could not provide a clear answer regarding their history of hepatitis A infection. Thus, a total of 2485 participants (who were confident in their infectious status) were included in the analysis of anti-HAV antibodies depending on the presence or absence of previous hepatitis A. Hepatitis A was reported by 69 volunteers, which accounted for 2.72% (95% CI: 2.1–3.4) of the total sample. Among these individuals, 58 were positive for anti-HAV IgG (84.1%, 95% CI: 73.7–90.9). In the subgroup of individuals who confidently denied having had HA (*n* = 2416, 95.4% of the entire sample, 95% CI: 94.5–96.2), anti-HAV IgG was detected in 441 people (18.3%, 95% CI: 16.8–19.8). The prevalence of anti-HAV IgG in different age groups among individuals with or without a history of HA is presented in Figure 3 (detailed data for all categories are provided in Table A1, Appendix A).

None of the volunteers reported a history of HEV infection. A definitive answer was not given by 28 individuals (1.1%, 95% CI: 0.7–1.6), among whom anti-HEV Abs were detected in 8 cases (28.6%, 95% CI: 13.2–48.7). In the subgroup of participants who confidently denied previous HEV exposure (*n* = 2505, 98.9% of the total sample, 95% CI: 98.4–99.3), anti-HEV Abs were found in 564 people (22.5%, 95% CI: 20.9–24.2).

Among the 68 individuals who reported having had HA, both anti-HAV and anti-HEV Abs were detected in 16 persons (23.5%, 95% CI: 14.1–35.4).

### 3.4. Seroprevalence of HAV and HEV by Field of Activity

**Figure 4:**
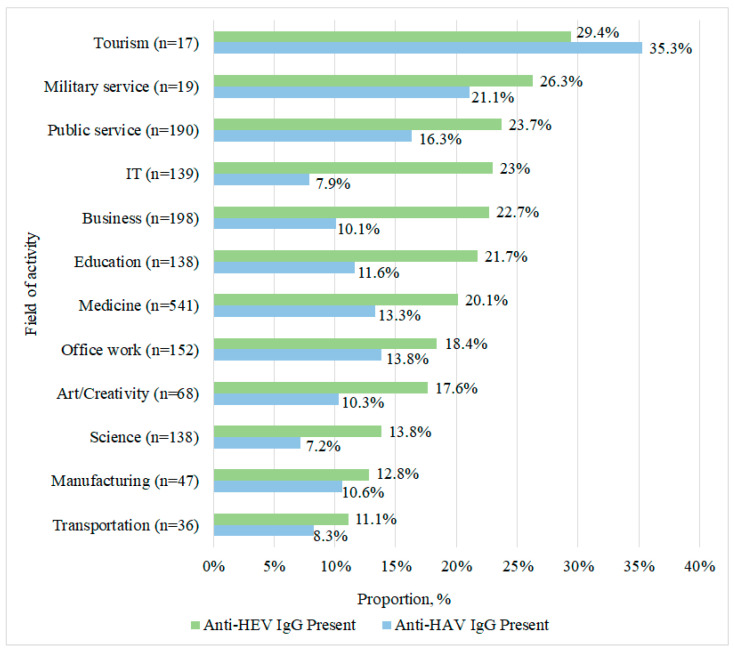
Prevalence distribution of anti-HAV and anti-HEV antibodies by professional category.

The significance of professional activity for both types of seroprevalence was assessed (Figure 4) (detailed data for all categories are provided in Table A2, Appendix A). When assessing seroprevalence by volunteer professional sector, a high frequency of anti-HEV Abs (50.0%, 95% CI: 6.8–93.2) and a low frequency of anti-HAV Abs (0%, 95% CI: 0.0–60.2) were found among agricultural workers. However, this group is extremely small in size (*n* = 4).

In the pensioner group, a high prevalence of both anti-HAV Abs (*n* = 248 out of 363, 68.3%, 95% CI: 63.4–72.9) and anti-HEV Abs (*n* = 148 out of 363, 40.8%, 95% CI: 35.8–45.9) was observed. In contrast, preschool children showed low seroprevalence (anti-HAV IgG *n* = 2 out of 16, 12.5%, 95% CI: 3.5–36.0; anti-HEV IgG *n* = 0 out of 16, 0%). Similarly, schoolchildren exhibited low values (anti-HAV IgG *n* = 7 out of 91, 7.7%, 95% CI: 3.8–15.0; anti-HEV IgG *n* = 6 out of 91, 6.6%, 95% CI: 3.1–13.6), as did students (anti-HAV IgG *n* = 3 out of 85, 3.5%, 95% CI: 1.2–9.9; anti-HEV IgG *n* = 7 out of 85, 8.2%, 95% CI: 4.0–16.0).

Among the working-age population, HEV seroprevalence was found to be higher than HAV in four groups (IT, business, education, healthcare). In the IT-sector, anti-HAV Abs were detected in *n* = 11 out of 139 individuals (7.9%, 95% CI: 4.5–13.6), while anti-HEV Abs were present in *n* = 32 out of 139 (23%, 95% CI: 16.8–30.7). This difference was significant with a chi-square of 11.004 at *p* = 0.0009, degrees of freedom (df) = 1, and an odds ratio (OR) of 3.480 (95% CI: 1.7–7.2). In the business sector, anti-HAV Abs were found in *n* = 20 out of 198 individuals (10.1%, 95% CI: 6.6–15.1), whereas anti-HEV Abs were found in *n* = 45 out of 198 (22.7%, 95% CI: 17.4–29.1). The difference here was also significant with a chi-square of 10.602 at *p* = 0.0011, df = 1, and an OR of 2.618 (95% CI: 1.5–4.6). In the education sector, anti-HAV Abs were found in *n* = 16 out of 138 individuals (11.6%, 95% CI: 7.3–18.0), but anti-HEV Abs were found in *n* = 30 out of 138 (21.7%, 95% CI: 15.7–29.3). This difference reached significance with a chi-square of 4.409 at *p* = 0.0358, df = 1, and an OR of 2.118 (95% CI: 1.1–4.1). In the healthcare sector, anti-HAV Abs were found in *n* = 72 out of 541 individuals (13.3%, 95% CI: 10.7–16.4), in contrast to anti-HEV Abs being present in *n* = 109 out of 541 (20.1%, 95% CI: 17.0–23.7). A significant difference exists here as well, characterized by a chi-square of 8.599 at *p* = 0.0034, df = 1, and an OR of 1.644 (95% CI: 1.2–2.3). No other groups showed statistically significant differences in the prevalence of anti-HEV or anti-HAV Abs.

High prevalence of both Abs (anti-HEV, anti-HAV) was noted in the groups related to tourism (anti-HAV Abs *n* = 6 out of 17, 35.3%, 95% CI: 17.3–58.7; anti-HEV IgG Abs *n* = 5 out of 17, 29.4%, 95% CI: 13.3–53.1), military personnel (anti-HAV IgG Abs *n* = 4 out of 19, 21.1%, 95% CI: 8.5–43.3; anti-HEV Abs *n* = 5 out of 19, 26.3%, 95% CI: 11.8–48.8), and public service employees (anti-HAV IgG Abs *n* = 31 out of 190, 16.3%, 95% CI: 11.7–22.2; anti-HEV Abs *n* = 45 out of 190, 23.7%, 95% CI: 18.2–30.2).

The lowest seroprevalence of both Abs (anti-HEV, anti-HAV) was found in transportation workers (anti-HAV Abs *n* = 3 out of 36, 8.3%, 95% CI: 2.9–21.8; anti-HEV Abs *n* = 4 out of 36, 11.1%, 95% CI: 4.4–25.3), manufacturing workers (anti-HAV Abs *n* = 5 out of 47, 10.6%, 95% CI: 4.6–22.6; anti-HEV Abs *n* = 6 out of 47, 12.8%, 95% CI: 6.0–25.2), and science workers (anti-HAV Abs *n* = 10 out of 138, 7.2%, 95% CI: 4.0–12.8; anti-HEV IgG Abs *n* = 19 out of 138, 13.8%, 95% CI: 9.0–20.5).

Among office workers, the prevalence of anti-HAV Abs was 13.8% (*n* = 21 out of 152, 95% CI: 9.2–20.2), while that of anti-HEV was 18.4% (*n* = 28 out of 152, 95% CI: 13.1–25.3). In art and creative workers, these values were 10.3% (*n* = 7 out of 68, 95% CI: 5.1–19.8) and 17.6% (*n* = 12 out of 68, 95% CI: 10.4–28.4), respectively.

### 3.5. Association Between HEV Seropositivity and History of Surgical Interventions or Blood Transfusions

Although the main route of transmission for both HAV and HEV is fecal–oral, there is evidence suggesting that HE can be transmitted through blood and organ transplantation. Some patients develop the disease after blood transfusion or surgery, especially in endemic regions with high infection prevalence. On the other hand, HA transmission via blood is extremely rare because the infection develops rapidly, and virus is rarely detected in significant amounts in patient blood. In light of this information, HEV seroprevalence was analyzed in relation to intervention history (surgery, blood transfusion) as shown in Table 2.

The study found significantly higher HEV Ab seroprevalence in the group of volunteers with a history of blood transfusion and/or surgical intervention (χ^2^ = 12.129 at *p* = 0.0005, df = 1, OR = 1.410, 95% CI: 1.2–1.7).

### 3.6. Hepatitis A Vaccination Coverage and Its Impact on Seroprevalence

Only 2384 individuals responded to the question about past vaccination in their medical history, of whom 44 were found to be vaccinated (1.8%, 95% CI: 1.4–2.5). All 44 vaccinated individuals had documented proof of vaccination (vaccination certificates or medical records). All these individuals were aged 12 years or older (Table 3). It is worth noting that only 12 out of the 44 vaccinated volunteers had detectable Abs, which corresponds to 27.3% (95% CI: 15.0–42.8). A total of 2244 volunteers reported no history of HA vaccination or previous HAV infection. Among them, anti-HAV Abs were detected in 409 individuals (18.2%, 95% CI: 16.7–19.9).

## 4. Discussion

### 4.1. Main Findings and Seroprevalence Overview

Before interpreting the findings, the sampling strategy should be considered. Participation was voluntary and web-based, leading to an overrepresentation of women (67.7%) and middle-aged adults (40–59 years), while children and young adults were underrepresented. These factors limit the generalizability of absolute seroprevalence estimates to the entire population of Belgrade, particularly for age groups most relevant to vaccination policies.

Over the course of the study, anti-HAV Abs were detected in 20.5% of individuals. This indicates that every fifth examined conditionally healthy individual either had a previous HA infection, underwent HA immunization, or experienced an undetected infection (i.e., asymptomatic or mild HA without significant clinical manifestations). A significantly lower frequency of anti-HAV Abs was found among volunteers who reported no history of HAV vaccination or HA itself (*n* = 2244), amounting to only 18.2%. This confirms previously unrecognized contact with HAV within a substantial portion of the studied cohort. It also highlights a high level of naturally acquired immunity among the general public, which reduces the likelihood of large-scale outbreaks.

### 4.2. HAV Epidemiology: Comparison with Other Countries and the Role of Vaccination

The overall anti-HAV IgG seroprevalence of 20.5% observed in our Belgrade study sample positions Serbia within the spectrum of countries transitioning from high to low hepatitis A endemicity. Our findings are remarkably consistent with data from the Czech Republic, where a large-scale study reported an anti-HAV seroprevalence of 21.1% [27]. This similarity likely reflects parallel improvements in sanitary conditions and living standards in Central Europe over recent decades. In contrast, the seroprevalence in our study sample is significantly higher than the 11.8% reported in a study of blood donors from west-central Poland [28]. This disparity may be attributed to differences in study populations, historical infection rates, or regional variations in vaccination policies and coverage. The contrast becomes even more pronounced when compared to non-European settings. For instance, a study in Rio de Janeiro, Brazil, found an overall HAV seroprevalence of 67.8% among special population groups [29]. This figure is more than three times higher than ours and is characteristic of regions where improvements in water safety and sanitation occurred more recently, leading to higher rates of exposure in childhood. This profile confirms Serbia’s successful transition to a low-endemicity pattern for HAV, driven by sustained improvements in hygiene and sanitation, rather than widespread vaccination.

Hepatitis A is not included among the infectious diseases for which mandatory routine immunization is implemented according to the Serbian national vaccination calendar [30]. Instead, national regulations define recommended (non-mandatory) vaccination for specific vulnerable population groups [31]. According to these recommendations, active immunization is carried out with an inactivated HA vaccine (administered as a single primary dose) in individuals with chronic liver disease, intravenous drug users, persons with a liver transplant, individuals working in poor hygienic conditions, and men who have sex with men (MSM). To achieve long-term protection, a second dose is recommended no earlier than six to twelve months after the first dose. However, despite these recommendations, the actual uptake of HA vaccination remains extremely low. In our study population, only 1.8% of participants reported receiving any dose of HAV vaccine. Vaccination is available in public health institutions at a price of about 46 EUR [32]. Prices in the private sector are significantly higher, reaching up to 79 EUR, although detailed data on private-sector availability remain limited.

Long-term studies from Germany [33], Belgium [34], China [35] and Taiwan [36] have shown that the two-dose inactivated HAV vaccine induces sustained immunity, with 95–100% of recipients maintaining protective anti-HAV IgG levels for extended periods. Across these cohorts, serological protection remains robust for at least 15–20 years, and modelling data predict persistence for two to three decades, supporting current recommendations against routine booster dosing.

The observation that only 12 of 44 vaccinated individuals (all with documented vaccination history) were anti-HAV IgG positive (27.3%) requires cautious interpretation. Critical information was not available for analysis: the number of doses received (one vs. two) and the time elapsed since vaccination. Without these variables, it is not possible to determine whether seronegativity reflects waning antibodies, incomplete primary immunization, or a true lack of vaccine response. Therefore, this finding should not be interpreted as evidence of poor vaccine efficacy, but rather as a data gap that limits conclusions about vaccine-induced immunity in this cohort.

Notwithstanding these data gaps, incomplete vaccination, irregular dosing, and/or natural waning of detectable Abs remain possible explanations for the observed seronegativity among vaccinated individuals. These patterns have also been reported in population studies from Turkey, China, and Brazil. Up to one third of individuals who self-report HAV vaccination in these settings had received only a single dose, which markedly reduces long-term seroconversion. Immunological evidence further shows that anti-HAV IgG levels may fall below detection 10–15 years after immunization despite preserved anamnestic responses and memory B-cell–mediated immunity. In other words, seronegativity does not necessarily indicate loss of vaccine-induced protection [34,37]. Serbia and comparable middle-income countries likely share similar structural factors known to reduce post-vaccination seropositivity or contribute additional variability: targeted rather than universal vaccination; fragmented delivery (central procurement with decentralized administration); and potential cold-chain vulnerabilities [38].

For context, a population-based study conducted in 2025 indicated that when comparing frequencies of anti-HAV Abs among vaccinated individuals, a stronger and longer-lasting immune response was observed following a double-dose vaccination regimen compared to single-dose administration [39]. Some studies show that Ab titers may decrease below the detection threshold over time while maintaining cellular immune memory, although this could be due to an incomplete vaccination course or inaccurate questionnaire data [40]. It remains undisputed that even after receiving the initial vaccination series, monitoring Ab levels and considering booster shots if necessary remain essential steps towards establishing robust and durable immune protection.

### 4.3. HEV Epidemiology: Local Context, Zoonotic Reservoir, and Transmission Routes

Currently, the HEV vaccine has not gained sufficient distribution globally, and no licensed vaccine is available in Serbia. Consequently, the immunity profile observed in our population reflects almost exclusively past natural infection, making seroprevalence studies a direct measure of historical exposure.

All anti-HEV IgG results were obtained using a single commercial ELISA kit without independent confirmation by an alternative method. Reported HEV seroprevalence is known to vary substantially depending on the assay used. Therefore, our estimates are assay-dependent and should be interpreted with this limitation in mind.

To interpret our findings, we first contextualized them within the global epidemiology of HEV. A comprehensive 2020 meta-analysis by Li et al., which synthesized data from 419 studies encompassing over 1.1 million individuals, estimated the global anti-HEV IgG seroprevalence at 12.5% [41]. This analysis revealed a distinct geographical gradient in HEV exposure. The highest seroprevalence was observed in Africa (21.8%, 95% CI: 13.1–32.0), followed by Asia (15.8%, 95% CI: 13.3–18.5). In Europe, the pooled seroprevalence was considerably lower at 9.3% (95% CI: 7.4–11.5). The Americas showed even lower estimates, with North America at 8.1% (95% CI: 5.5–11.1) and South America at 7.3% (95% CI: 4.8–10.2). The lowest seroprevalence was reported in Oceania at 6.0% (95% CI: 1.2–14.0) [41]. The anti-HEV IgG seroprevalence of 22.6% detected in our Belgrade study sample is more than double the European average (9.3%) and, in fact, exceeds the pooled estimates for all world regions except Africa. This positions Serbia among the higher-endemicity areas for HEV on a global scale. This figure is particularly striking when compared to earlier estimates from Serbia. The same meta-analysis by Li et al. included two Serbian studies conducted prior to 2020, involving a total of 726 participants, which reported a pooled seroprevalence of only 8.5% [41]. The discrepancy between our findings and these previous estimates is likely multifactorial. Key differences include study design and population selection, sample size (our single study sample of 2533 volunteers provides greater statistical power compared to the smaller, pooled sample of 726 from two separate studies), geographic coverage (our study focused on the capital city, which may differ from other regions of Serbia included in earlier work), and potentially the sensitivity and specificity of the diagnostic assays used. These methodological differences preclude direct comparisons and highlight the need for standardized seroepidemiological surveys to accurately monitor HEV trends over time.

Among all hepatitis E viruses, only those from Orthohepevirus genotypes 1, 2, 3, 4, and 7 are contagious to humans. Genotypes 1 and 2 are mainly transmitted through water and are major causes of acute HE outbreaks in developing countries. In contrast, genotypes 3, 4, and 7 are zoonotically transmitted, including by consumption of infected animal products [42]. This usually results in sporadic cases of HE both in developed and developing countries such as Serbia [43].

The HEV Ab seroprevalence detected in our study (22.6%) is lower than values obtained in Polish research (49.6%, *n* = 246) [44]. It is also lower than values found in Corsica (France) (55.8%, *n* = 2705) [45]. According to Grabarczyk et al., anti-HEV IgG seroprevalence varied between 22.7% and 60.8% in different age groups (18–27 and 48–57 years old, respectively) [46]. Like our findings regarding HEV Ab seroprevalence, a retrospective study conducted in neighboring Bulgaria showed a seroprevalence of 18.1% (*n* = 773) [47]. Another study published in 2019 included 555 blood donors and reported a seroprevalence of 25.9% among examined Polish donors [48].

Collectively, these comparisons confirm that HEV seroprevalence in Belgrade (22.6%) is among the higher ranges reported in Europe, though still below the peaks observed in Poland and France. This elevated burden requires a biological explanation rooted in local exposure patterns.

Available evidence suggests that the high HEV seroprevalence in Serbia (22.6%) is indicative of long-standing zoonotic circulation of genotype 3 in the swine reservoir and continuous low-intensity exposure of humans [49]. Serological studies from Serbia have revealed high HEV IgG prevalence among domestic pigs at around 55% in commercial and 75% in backyard herds, as well as around 52% in wild boars [50], with phylogenetic investigations confirming close relatedness between human and animal strains. HEV RNA has been identified in ≈29% of pig livers at slaughter [12], confirming that infected animals frequently enter the human food chain. National dietary surveys indicate that Serbian adults consume ≈166 g/day of meat and meat products, with pork as the dominant red meat (≈16 kg per capita/year) and a particularly high intake of dry-fermented sausages (~56 g/day), bacon, and smoked meats. Widespread use of traditional pork products, in particular dry-fermented and smoked products which may have incomplete heat treatment, provides biologically plausible conditions for foodborne HEV genotype 3 infection [51]. Although systematic data on HEV RNA in Serbian meat products are not yet available, extensive European data implicate pork and liver-derived products as recognized routes of transmission. Altogether, high viral circulation in swine, regional heterogeneity in human seroprevalence, sustained dietary exposure, and occupational contact factors provide a logical explanation for Serbia’s seroepidemiological profile and the age-related accumulation of subclinical HEV infections. However, because individual-level dietary, travel, and behavioral exposure data were not collected in this study, these interpretations remain hypothetical. Confirmatory studies with detailed exposure questionnaires and, where feasible, HEV RNA testing in locally available meat products are required to establish causal pathways.

Furthermore, 7.1% of individuals tested positive for Abs against both HAV and HEV. This suggests either exposure to two different hepatitis viruses at separate times or comorbid infections in their medical histories. In addition to foodborne and zoonotic routes, the potential for parenteral transmission of HEV deserves consideration. The study found a significantly higher occurrence of anti-HEV Abs among participants with a history of blood transfusion and/or surgical procedures. Although HEV is usually considered a zoonotic, foodborne infection, accumulating European data show that parenteral transmission through asymptomatic, viremic donors is well documented [52,53,54]. In Serbia, routine HEV screening of donated blood, organs, or tissues is not performed, insofar as mandatory donor testing is limited to HIV, HBV, HCV, and syphilis [55]. HEV testing of donors is carried out only in exceptional situations, most commonly: when liver enzymes are unexpectedly elevated; when clinical signs suggest acute hepatitis; or when transplantation is intended for a severely immunocompromised recipient. In routine practice, individuals who report such symptoms would typically be considered unsuitable for blood donation. However, the major challenge lies in the subclinical or asymptomatic course of HEV infection, which allows potentially infectious donors to pass standard eligibility screening.

Several factors likely contribute to this trend, including changes in gut microflora, and/or disruption of protective mucosal barriers in the gastrointestinal tract, facilitating entry of HEV into the body. Postoperative periods are commonly characterized by suppressed immunity, increased inflammation, and overall strain on the body. These may lead to activation of latent infections or dormant viruses. Additionally, post-operative medications may have suppressive effects on the immune system [56,57,58]. Collectively, these factors offer a biologically plausible hypothesis for the observed association, but they do not constitute proof of causation. Furthermore, the cross-sectional design and reliance on self-reported medical history preclude causal conclusions from this association. This finding should be interpreted as a correlation that may reflect multiple underlying mechanisms, including parenteral exposure, shared risk factors (e.g., more frequent healthcare contact for other conditions), or reverse causality. Therefore, these results generate a hypothesis rather than provide direct evidence for transfusion- or procedure-related HEV transmission.

Although nosocomial HEV transmission is exceedingly rare, a German investigation confirmed patient-to-patient spread after two ICU patients consecutively occupied the same bed space, with identical viral sequences detected [59]. The second, severely immunosuppressed patient developed chronic HEV, and no further cases occurred, indicating that such events require both environmental lapses and marked host immunosuppression.

### 4.4. Demographic Determinants: Age and Gender

Comparative analysis based on gender revealed higher Ab seroprevalence for hepatitis A and E viruses among men compared to women. However, statistically significant differences were confirmed only for HA. A similar trend has been noted in the previously mentioned meta-analysis [41]. This pattern may be explained by a combination of behavioral and occupational factors. Men are traditionally more often employed in sectors with an increased risk of fecal–oral transmission, such as construction, infrastructure maintenance, or agriculture. They may also be more frequently exposed through eating outside the home or during travel, which predominantly affects HA transmission. Regarding HEV, although the trend was similar, statistical significance was not reached. This may indicate that the main routes of HEV transmission in the studied population (e.g., foodborne) are less gender-dependent, or it may reflect a smaller effect size requiring a larger sample for detection. Thus, the confirmed differences for HAV underscore the importance of considering gender-specific risks when planning prevention programs. However, because relevant data (e.g., occupation-specific hygiene practices, travel, dietary habits) were not collected in this study, this interpretation remains speculative and requires further investigation. The nature of the association for HEV requires further study.

Although humanity has known about catarrhal jaundice for quite some time, it was not until 1888 when S.P. Botkin first hypothesized its infectious nature. The modern term “hepatitis A” did not enter common usage until 1947, thanks to F. McCollum. Based on historical evidence, one can infer that the sharp decline in HAV Ab seroprevalence in populations under 50 years old might be associated with the discovery of the hepatitis A virus in 1973 [60].

The first vaccines against HA were developed in the 1990s. In 1992, the “Havrix” vaccine [61] became the first licensed product, followed by its market release in 1995 [62]. Currently, there are over four commercially available HA vaccines, including Havrix, Vaqta, Avaxim, Algovak M, and others. However, the lowest rate of HAV Ab detection, only 2.8%, was observed in the age group 18–29 years. This could potentially be linked to the period during which the first accessible HA vaccines were introduced.

A historical overview of HA in Serbia features a clear transition from high endemicity to a low-endemicity profile, driven largely by improvements in water safety, sanitation, and household hygiene consistent with global patterns described in transitioning countries [38]. Over the past several decades, gradual expansion of Serbia’s water-supply and wastewater infrastructure has played a central role in reshaping the epidemiological landscape. Although precise historical documentation of rural infrastructure development is limited, WHO/UNICEF Joint Monitoring Programme data show a steady rise in access to improved drinking-water sources, exceeding 98% of the population. This has occurred alongside ongoing modernization of sewerage systems since the early 2000s [63,64,65]. Despite these advances, sanitation disparities persist in rural communities, where reliance on aging or unregulated septic tanks continues to facilitate localized fecal–oral transmission.

Historical surveillance data from the Autonomous Province of Vojvodina illustrates the formerly high HAV endemicity. Between 1988 and 2009, more than 10,000 cases were reported, accompanied by four epidemic waves at intervals of 3–6 years. This cyclical endemic-epidemic pattern reflects sustained past community transmission. In contrast, recent national data show a sustained decline in HAV incidence (particularly among children) indicating reduced early-life exposure after improvements in water, sanitation, and food hygiene. The disappearance of epidemic cycles and a shift of cases toward adults confirm Serbia’s transition to a very low-endemic setting, although persistent rural infrastructural gaps still require targeted public-health measures.

As far as HE is concerned, we currently lack information regarding the prevalence of this disease due to various reasons. In our research, we identified a correlation between HE seroprevalence and participant age. Antibodies against HEV persist for approximately 20–30 years [66,67]. Insofar as hygienic conditions have generally improved over time, lower seroprevalence in younger groups is fully expected. However, in light of Ab persistence for 2 or 3 decades, it is still unclear if the higher values in older groups are related to repeated lifelong exposures or enduring immunity formed in the era before various improvements.

Regarding HAV, there was indeed a correlation with age groups above 30 years old. In younger groups below 30, HAV Ab prevalence was low. It can be assumed that the younger generation grew up in improved sanitary-hygienic conditions, including access to clean water and compliance with hygienic standards, which reduced the risk of HAV infection. It is also important to note that the first HA vaccines were developed about 30 years ago, which likely contributed to reducing outbreaks.

It should be noted, however, that the R^2^ values presented in Figure 2 are derived from aggregated age-group data and are presented for descriptive illustration only; they may not reflect individual-level variability and may overstate the strength of the age–seroprevalence relationship.

Only 14 out of 2533 surveyed volunteers reported having a history of HA or HE. However, all mentioned cases referred specifically to HA, even though three of them tested positive for both anti-HAV and anti-HEV Abs. Moreover, 2403 volunteers confidently denied any prior experience with HE, yet results showed that 22.5% of them carried HEV Abs, indicating past exposure to the virus and/or possibly silent HE infection.

These findings underscore the need for new diagnostic approaches concerning viral hepatitis in Serbia. Efforts should focus on several areas: raising awareness among healthcare professionals regarding symptoms and risks associated with hepatitis A and E viruses; implementing advanced diagnostic tests; and training staff in identifying specific markers of infection. Additionally, prevention programs targeting the general population must be enhanced, incorporating educational campaigns and hygienic measures aimed at preventing transmission via contaminated food and water. Such comprehensive initiatives will significantly reduce the burden of viral hepatitis and bring the country closer to achieving WHO goals for eliminating viral hepatitis by 2030.

### 4.5. Occupational and Behavioral Risk Factors

When analyzing Ab seroprevalence (anti-HAV, anti-HEV) among employed individuals across different professional sectors, four groups stood out: the IT sector, the business sphere, education, and medicine. Notably, HEV Ab seroprevalence was significantly higher compared to HAV Ab seroprevalence in these groups. The following interpretations are exploratory and hypothesis-generating, as this study did not directly assess dietary habits, travel history, or specific occupational exposures.

Among the four specified groups, the highest HAV seroprevalence was found among healthcare workers. This can be associated with occupational exposure to patient biological fluids, medical instruments, and equipment. For comparison, in a local Russian population study (St. Petersburg and the Leningrad region), anti-HAV Ab seroprevalence was 39.1% and anti-HEV was 5.4% [39,68]. In a Kyrgyz study (*n* = 1276), these values were 93.8% and 7.8%, respectively [69]. The elevated HAV seroprevalence observed among healthcare workers may also reflect broader population-level factors such as cohort effects and background epidemiology that are not primarily related to occupational risk. Healthcare personnel in Serbia predominantly belong to age groups that experienced high early-life HAV transmission. This is consistent with regional data from St. Petersburg [39], Kyrgyzstan [69], Turkey [70], and Iran [71], where anti-HAV IgG seropositivity closely paralleled age-specific population trends rather than healthcare-related risk. Regarding HEV, evidence from Germany [72], the Netherlands [73], and Egypt [74] shows that nosocomial transmission is negligible and that HEV genotype 3 infection in Europe is overwhelmingly driven by foodborne and zoonotic pathways.

However, although this factor is important, it does not determine the high level of HEV seropositivity among representatives of the three other listed fields of activity. Beyond occupational determinants, susceptibility to HAV and HEV in Serbia is further influenced by individual behavioral and hygiene-related factors. Notably, multiple European studies report elevated HAV and HEV seroprevalence among MSM [75], reflecting sexual practices that facilitate fecal–oral transmission independent of foodborne exposure. Although our study did not collect data on sexual behavior, published evidence from Japan and South Korea confirms that MSM remain a risk group for HAV infection in Asia as well [76,77]. Other behavioral influences, such as inconsistent hand hygiene, food-safety practices, and dietary habits, play a secondary but contributory role in shaping risk.

Differences in seroprevalence across occupational groups reflect distinct exposure pathways defined by epidemiological models of foodborne and fecal–oral transmission. In tourism workers (HEV 29.4%, HAV 35.3%) and military personnel (26.3%, 21.1%), the risk–exposure stratification model applies. These groups frequently consume buffet-style, mass-catered, and travel-related meals. These settings have repeatedly been shown to carry higher HEV/HAV contamination risk in European outbreak investigations. Public service employees (HEV 23.7%, HAV 16.3%) fit the source–pathway–receptor model, wherein irregular schedules, high interpersonal contact, and dependence on externally prepared food increase the number of exposure pathways, even without international mobility.

Transportation, production, and scientific workers (HEV 11–14%, HAV 7–11%) align with the exposure heterogeneity model, wherein stable daily routines, minimal foreign travel, and predominant reliance on home-prepared meals restrict exposure windows to both viruses. By contrast, IT, business, and education workers (HEV 21–23%, HAV 8–12%) fit the behavioral risk-gradient model, wherein higher socioeconomic status is associated with greater consumption of diverse pork products (including dry-fermented, smoked, undercooked meats) and long-distance travel. Both factors are strong predictors of HEV genotype 3 exposure in European cohorts [78].

The pattern of consistently higher HEV than HAV across all occupations reflects the dual-pathway model, wherein HAV declined with sanitation improvements, while HEV persists due to entrenched zoonotic transmission from pork products. Direct testing of this hypothesis would require targeted exposure assessment.

Given that HE often presents with mild clinical symptoms and remains asymptomatic in nearly half of cases, the absence of pronounced signs diminishes interest in laboratory testing. Consequently, assessing the true extent of infection becomes challenging. Therefore, addressing the issue of HE infectious load remains critical, necessitating heightened public awareness and improved preventative measures by authorities.

### 4.6. Study Limitations

This study has several limitations. Its cross-sectional design does not allow for the establishment of cause-and-effect relationships or the analysis of infection dynamics over time. First, concerning information bias, key variables were primarily collected via self-reporting (including vaccination status, hepatitis A disease). For participants who reported vaccination or past disease, this information was verified against vaccination certificates or medical records where available. However, for those who denied vaccination or disease, no independent verification was possible. This approach is inherently susceptible to recall bias, particularly among older adults who may not accurately remember vaccinations or past subclinical infections. Second, the study did not collect detailed data on specific behavioral risk factors beyond intervention history (surgery, blood transfusion). Data on sexual behavior, including MSM status, were not collected. This limits our ability to assess the contribution of sexual transmission to the observed gender differences in HAV and HEV seroprevalence. A lack of information on participant travel history limits the ability to analyze the contribution of imported infections.

The most significant methodological limitation concerning HE is the use of ELISA alone without confirmation by an alternative method. Given the known variability in the specificity of commercial anti-HEV IgG ELISA tests, verification of positive results using a more specific method (e.g., recombinant immunoblot assay) would have improved the reliability of the HEV seroprevalence data.

Finally, sample size and representativeness present notable constraints. The voluntary nature of participation, and enrollment via a web application, likely introduced a selection bias, constituting non-probabilistic (convenience) sampling, as evidenced by the overrepresentation of female and middle-aged participants. This may limit the generalizability of absolute prevalence estimates to the entire population of Belgrade. The voluntary, web-based recruitment did not allow us to reach the target number of children and adolescents. The small sample sizes in pediatric groups increase the uncertainty around seroprevalence estimates in these critical age cohorts. Therefore, conclusions regarding population immunity profile in these younger age groups, which are central to vaccination policy, should be drawn with particular caution. When seroprevalence is zero in a small age group (e.g., anti-HEV in children aged 1–5 years), the Clopper–Pearson confidence intervals are wide (0.0–24.7). These wide intervals reflect the small sample size and should not be interpreted as precise estimates; they indicate high uncertainty rather than a true zero population seroprevalence. Despite these limitations, this study provides valuable, population-based serological data that reflect the changing epidemiology of HAV and HEV infections over several decades in Belgrade, largely driven by natural infection in the absence of widespread vaccination.

## 5. Conclusions

Bearing in mind the assay-dependent nature of the anti-HEV estimates and the sampling limitations discussed above, the data collected indicate substantial levels of antibodies against hepatitis A and E viruses among the studied sample of Belgrade residents. Addressing this issue requires an interdisciplinary approach encompassing preventive vaccination efforts, expansion of diagnostic capabilities, and educational campaigns focused on improving hygiene and prevention of infection. It is recommended to conduct further studies to identify risk factors and pathways of infection transmission. Continued surveillance of public health, and implementation of preventive measures, are crucial. Developing effective strategies can slow the growth of disease rates and ensure achievement of WHO objectives aimed at reducing global burdens of viral hepatitis by 2030.

## Figures and Tables

**Table 1:** Seroprevalence of anti-HAV and anti-HEV antibodies by age group.

Age Group (Years)	Number of Volunteers	Anti-HAV IgG Ab Positive		Anti-HEV IgG Ab Positive	
		*n*	%, 95% CI:	*n*	%, 95% CI:
1–17	118	10	8.5% (4.7–14.9) #	5	4.2% (1.8–9.5) #
1–5	13	1	7.7% (1.4–33.3)	0	–
6–11	43	4	9.3% (3.7–21.6)	1	2.3% (0.4–12.1) #
12–17	62	5	8.1% (3.5–17.5) #	4	6.5% (2.5–15.4) #
18–29	249	7	2.8% (1.4–5.7) #	24	9.6% (6.6–13.9) #
30–39	501	29	5.8% (4.1–8.2) #	63	12.6% (10.0–15.8) #
40–49	688	51	7.4% (5.7–9.6) #	161	23.4% (20.4–26.7)
50–59	468	102	21.8% (18.3–25.8)	121	25.9% (22.1–30.0)
60–69	320	172	53.8% (48.3–59.1) *	105	32.8% (27.9–38.1) *
70+	189	148	78.3% (71.9–83.6) *	93	49.2% (42.2–56.3) *
Total	2533	519	20.5% (18.9–22.1)	572	22.6% (21.0–24.3)

Note: * significantly higher than the total value; # significantly lower than the total value; *p* < 0.05. Ab, antibody. “–“ indicates not applicable (no individuals in the denominator).

**Table 2:** HEV Ab seroprevalence by intervention history (surgical, blood transfusion).

History of Surgical Interventions and/or Blood Transfusions	N	Anti-HEV IgG Ab Positive		
		*n*	%	95% CI:
Yes	1185	304	25.7 *	23.2–28.2
No	1246	245	19.7	17.6–22.0
Total:	2431	549	22.6	21.0–24.3

Note: * significantly higher compared to the “No” group, *p* < 0.05.

**Table 3:** Hepatitis A virus (HAV) vaccination coverage by age group.

Age Group, (Years)	Number of Volunteers	Vaccinated Against HAV		Anti-HAV IgG Seropositivity Among Vaccinees	
		*n*	% (95% CI)	*n*	% (95% CI)
1–5	12	0	–	0	–
6–11	40	0	–	0	–
12–17	62	3	4.8 (1.7–13.3)	1	33.3 (0.8–90.6)
18–29	232	6	2.6 (1.2–5.5)	2	33.3 (4.3–77.7)
30–39	467	10	2.1 (1.2–3.9)	3	30.0 (6.7–65.2)
40–49	647	13	2.0 (1.2–3.4)	1	7.7 (0.2–36.0)
50–59	442	9	2.0 (1.1–3.8)	5	55.6 (21.2–86.3)
60–69	306	2	0.7 (0.2–2.4)	0	–
70+	176	1	0.6 (0.1–3.1)	0	–
Total:	2384	44	1.8 (1.4–2.5)	12	27.3 (15.0–42.8)

Note: “–” indicates not applicable (no individuals in the denominator).

## Data Availability

The original contributions presented in this study are included in the article. Further inquiries can be directed to the corresponding author.

## Appendix A

**Table A1:** Anti-HAV IgG seroprevalence by age and hepatitis A history.

Age Group, Years	N Status Known	Anti-HAV IgG				Anti-HAV IgG			
		N (Had HA)	*n*	%	95% CI	N (Did Not Have HA)	*n*	%	95% CI
1–17	118	0	0	–	–	118	10	8.5	4.7–14.9
1–5	13	0	0	–	–	13	1	7.7	1.4–33.3
6–11	43	0	0	–	–	43	4	9.3	3.7–21.6
13–17	62	0	0	–	–	62	5	8.1	3.5–17.5
18–29	248	1	0	–	–	247	7	2.8	1.4–5.7
30–39	494	6	4	66.7	30.0–90.3	488	24	4.9	3.3–7.2
40–49	673	10	7	70	39.7–89.2	663	42	6.3	4.7–8.5
50–59	461	22	18	81.8	61.5–92.7	439	82	18.7	15.3–22.6
60–69	308	14	13	92.9	68.5–98.7	294	150	51	45.3–56.7
70+	183	16	16	100	80.6–100.0	167	126	75.4	68.4–81.4
Total	2485	69	58	84.1	73.7–90.9	2416	441	18.3	16.8–19.8

Note: “–” indicates not applicable (no individuals in the denominator).

**Table A2:** Hepatitis A and E viral markers by field of activity.

Field of Activity	N	Anti-HAV IgG Positive			Anti-HEV IgG Positive		
		*n*	%	95% CI	*n*	%	95% CI
Medicine	541	72	13.3	10.7–16.4	109	20.1	17.0–23.7
Science	138	10	7.2	4.0–12.8	19	13.8	9.0–20.5
Business	198	20	10.1	6.6–15.1	45	22.7	17.4–29.1
Education	138	16	11.6	7.3–18.0	30	21.7	15.7–29.3
Art/creativity	68	7	10.3	5.1–19.8	12	17.6	10.4–28.4
Manufacturing	47	5	10.6	4.6–22.6	6	12.8	6.0–25.2
Transport	36	3	8.3	2.9–21.8	4	11.1	4.4–25.3
Military service	19	4	21.1	8.5–43.3	5	26.3	11.8–48.8
Public service	190	31	16.3	11.7–22.2	45	23.7	18.2–30.2
Office work	152	21	13.8	9.2–20.2	28	18.4	13.1–25.3
Unemployed	112	18	16.1	10.4–24.0	23	20.5	14.1–28.9
Preschooler	16	2	12.5	3.5–36.0	0	–	–
Miscellaneous	179	35	19.6	14.4–26.0	46	25.7	19.9–32.6
Schoolchild	91	7	7.7	3.8–15.0	6	6.6	3.1–13.6
Student	85	3	3.5	1.2–9.9	7	8.2	4.0–16.0
Retiree	363	248	68.3	63.4–72.9	148	40.8	35.8–45.9
Tourism	17	6	35.3	17.3–58.7	5	29.4	13.3–53.1
Agriculture	4	0	–	–	2	50	15.0–85.0
IT	139	11	7.9	4.5–13.6	32	23	16.8–30.7
Total	2533	519	20.5	19.0–22.1	572	22.6	21.0–24.3

Note: “–” indicates not applicable (no individuals in the denominator).
